# Secular trends in physical fitness and nutritional status among Tibetan children and adolescents from 2005 to 2019: a secondary analysis of four CNSSCH surveys

**DOI:** 10.3389/fnut.2026.1891556

**Published:** 2026-09-30

**Authors:** Meng Yuan, Feng Peng, Bowen Song, Chenyu Liu, Xiaotong Zhang, Ming Li, Shuangshuang Sun, Desheng Li, Zhengwen Chen, Yi Sun

**Affiliations:** 1 School of Physical Education, Ludong University, Yantai, Shandong, China; 2 School of Physical Education, Tibet University for Nationalities, Xianyang, Shaanxi, China; 3 Xiguan Primary School, Yantai, Shandong, China; 4 School of Physical Education, University of Jinan, Jinan, Shandong, China

**Keywords:** children and adolescents, nutritional status, physical fitness, secondary analysis, Tibetan

## Abstract

**Objective:**

This study aimed to assess the long-term trends in physical fitness and their relationship with nutritional status among children and adolescents in Tibet from 2005 to 2019.

**Methods:**

This secondary analysis initially included 9,818 Tibetan children and adolescents aged 7–18 years from four waves of the Chinese National Survey on Students’ Constitution and Health (CNSSCH), conducted in 2005, 2010, 2014, and 2019. After data cleaning, 9,237 participants were retained for the final analysis. Statistical analyses included one-way analysis of variance (ANOVA) for continuous variables and chi-square tests for categorical variables to compare physical fitness indicators and nutritional status across survey years, supplemented by trend analyses. Fractional polynomial regression was employed to assess the relationship between Physical Fitness Index (PFI) and body mass index Z-score (BMI Z-score). Additionally, generalized additive models (GAMs) were used to assess the associations between PFI and adverse nutritional status (thinness, overweight, and obesity), with results expressed as odds ratios (*ORs*).

**Results:**

From 2005 to 2019, the PFI increased by 4.91 units among Tibetan children and adolescents, accompanied by an initial increase followed by a decline in the proportion of thinness, whereas overweight/obesity increased over the study period. Fractional polynomial regression revealed an inverted U-shaped association between BMI Z-score and PFI, with physical fitness being lower in children and adolescents with thinness or overweight/obesity than in those with normal weight. Further analyses based on prevalence and ORs showed that, as PFI decreased, the prevalence and odds of thinness increased more sharply than those of overweight/obesity. Using PFI values of −5 and 5 as representative examples, the estimated ORs for thinness at PFI = −5 ranged from 1.53 to 1.88 across survey years, whereas the corresponding ORs for overweight/obesity ranged from 1.10 to 1.75; at PFI = 5, the corresponding ORs declined to 0.16–0.43 for thinness and 0.49–0.90 for overweight/obesity.

**Conclusion:**

Although physical fitness improved among Tibetan children and adolescents over the study period, both thinness and overweight/obesity were associated with less favorable physical fitness. These findings suggest that maintaining an appropriate weight status may be important for promoting favorable physical fitness development among children and adolescents.

## 1 Introduction

Physical fitness refers to the body’s integrated functional capacities displayed during physical activity. The development of physical fitness in children and adolescents exerts a profound influence on their healthy growth and long-term well-being (1–3). Studies have demonstrated that high levels of physical fitness are associated with enhanced cognitive function, psychological well-being, and academic performance (4–7), whereas inadequate fitness correlates with an increased risk of chronic disease, and this elevated risk may persist into adulthood (8–10). Over the past few decades, there has been a pronounced global decline in the physical fitness of children and adolescents (11). Likewise, the fitness levels of Chinese youth have shown a substantial downward trajectory across successive cohorts (12).

The Physical Fitness Index (PFI), as a composite metric for evaluating overall fitness status, has seen increasingly widespread application (13). In recent years, several studies have provided in-depth analyses of the association between PFI and nutritional status in children and adolescents (14–16). For example, a bi-national study of Chinese and Japanese children and adolescents demonstrated an inverted U-shaped association between body mass index (BMI) and the PFI. Moreover, as BMI increased, the decline in PFI at higher BMI values was significantly steeper in Chinese boys than in their Japanese counterparts (17). Another study of Taiwanese children and adolescents revealed a nonlinear, age-dependent association between BMI and the PFI. During puberty, this association assumed a parabolic form, with the apex of the curve appearing sharper in boys than in girls (13). Although previous studies have extensively examined the association between physical fitness and nutritional status among children and adolescents, evidence regarding this association in populations living in distinctive geographical environments remains relatively limited.

Children and adolescents living in the Tibet Autonomous Region are exposed to a distinctive high-altitude environment characterized by chronic hypobaric hypoxia and other climatic and environmental conditions. Such environmental exposure during growth and development may influence physiological adaptation and multiple dimensions of physical fitness, potentially resulting in fitness profiles that differ from those observed among children and adolescents living at lower altitudes (18, 19). Previous studies have reported differences in physical fitness between Tibetan children and adolescents and their counterparts living in low-altitude regions (20, 21), highlighting the need to examine physical fitness in this population separately. With ongoing socioeconomic transition, the nutritional profile of Tibetan children and adolescents has also shifted, with declining undernutrition alongside increasing overweight and obesity (22). However, previous studies among Tibetan children and adolescents have largely relied on cross-sectional designs, whereas evidence on long-term changes in overall physical fitness and its relationship with nutritional status remains limited.

Therefore, using data from four waves of the Chinese National Survey on Students’ Constitution and Health (CNSSCH) conducted in 2005, 2010, 2014, and 2019, this study aimed to assess long-term trends in physical fitness and nutritional status among Tibetan children and adolescents and to examine the association between physical fitness and nutritional status across survey years.

## 2 Methods

### 2.1 Research design and participants

For this secondary analysis, data were obtained from four waves (2005, 2010, 2014, and 2019) of the CNSSCH, a nationally organized school-based survey conducted periodically under standardized sampling, measurement, and quality-control procedures. The CNSSCH adopted a stratified random cluster sampling design and followed standardized national procedures across survey waves (23–26). The present study analyzed the Tibetan subset of the CNSSCH database. Tibetan students included in this analysis were recruited from nationally designated survey sites located mainly in and around Lhasa, at an altitude of approximately 3,600 m. The participating schools were designated testing sites within the national CNSSCH survey framework. A total of 9,818 Tibetan children and adolescents aged 7–18 years who participated in the four selected CNSSCH survey waves were initially eligible for inclusion. Participants were excluded if they had missing data on height, weight, or any physical fitness test item required for calculation of the PFI, or if implausible values exceeding ±3 standard deviations were identified for the relevant anthropometric or physical fitness variables. After data cleaning, 9,237 participants were retained for the final analysis, including 4,558 boys and 4,679 girls (Figure 1). The final analytic sample comprised 2,275 participants in 2005, 2,544 in 2010, 2,297 in 2014, and 2,121 in 2019. Both urban and rural students were included in the Tibetan subset; however, their distribution was not balanced across survey waves. Earlier survey waves were mainly sampled from Lhasa, whereas the later waves also remained predominantly urban, with relatively limited rural representation. Therefore, separate urban–rural trend analyses were not performed because the urban and rural subsamples were not sufficiently balanced or comparable across survey years. The study protocol was approved by the Human Research Ethics Committee of East China Normal University (Approval No. HR 0077-2020). Written informed consent was obtained from all participants and their legal guardians prior to data collection in the original CNSSCH surveys. The use of the Tibetan CNSSCH data in the present study was authorized by the Education Department of the Tibet Autonomous Region.

**Figure 1:**
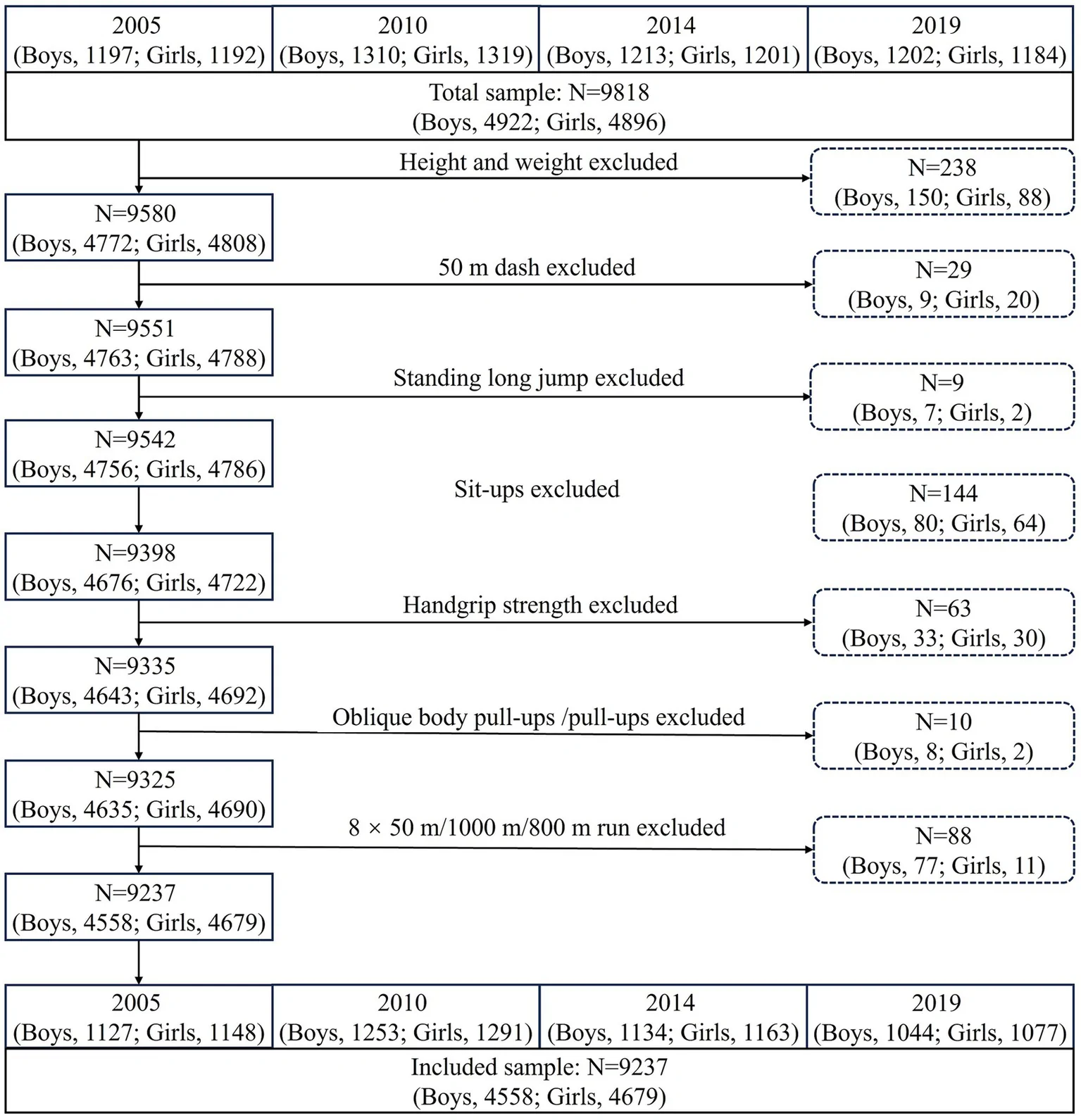
Flowchart of sample inclusion.

### 2.2 Physical fitness test

All anthropometric and fitness-related measurements in the CNSSCH were collected under unified survey procedures by qualified examiners (23). Equipment was checked and calibrated before use, and the same testing framework was applied across survey waves to promote consistency and reduce potential measurement variability. Anthropometric and physical fitness indicators included height, weight, forced vital capacity (FVC), standing long jump, sit-and-reach, handgrip, 50-meter dash, body muscular strength, and endurance running. Height was measured to the nearest 0.1 cm using a portable stadiometer (model TZG, Jiangyin No. 2 Medical Equipment Factory, Jiangsu, China), and body weight was measured to the nearest 0.1 kg using a lever-type scale (model RGT-140, Wuxi Weighing Apparatus Factory Co., Ltd., Wuxi, China). FVC was measured using an HJ20-SF-I electronic spirometer (measurement range: 0–9,999 mL; indicated accuracy: ≤ ± 5% F. S.) in accordance with the National Student Physical Health Standard (Trial Scheme) of China. Lower limb power was evaluated with the standing long jump, and flexibility was assessed using the sit-and-reach test. Body muscular strength was assessed by oblique body pull-ups in boys aged 7–12 years, standard pull-ups in boys aged 13–18 years, and 1-min sit-ups in girls aged 7–18 years. Handgrip strength was measured using a grip dynamometer accurate to 0.1 kg. Participants performed the test with the dominant hand, and the maximum value was recorded according to the standardized CNSSCH testing procedures. Speed was measured using the 50-meter dash, whereas endurance performance was assessed using the 8 × 50-meter shuttle run in children aged 7–12 years and distance endurance running (1,000 m for boys and 800 m for girls) in adolescents aged 13–18 years. The sample size and proportion of participants tested for each item are presented in the Supplementary Table S1.

Anthropometric indicators (height, weight, and BMI) and physical fitness measures were standardized separately by sex and single-year age using the corresponding median and standard deviation from the 2005 survey as the reference (Supplementary Tables S2, S3). For each indicator, the standardized score was calculated as:

$$Z=\frac{X-M_{2005}}{SD_{2005}}$$

where X represents the individual measurement, $M_{2005}$ represents the sex- and age-specific median of the corresponding indicator in the 2005 survey, and $SD_{2005}$ represents the corresponding sex- and age-specific standard deviation. The same 2005 reference parameters were applied to the 2010, 2014, and 2019 survey data to ensure comparability across survey waves. The 2005 survey was selected as the fixed reference because it was the earliest survey wave included in the present analysis, providing a common baseline for comparisons across different survey years. Nutritional status was classified according to BMI-for-age Z-scores based on the World Health Organization (WHO) Growth Reference for children and adolescents aged 5–19 years. Participants were categorized as thinness (BMI-for-age Z-score < −2), normal weight (≥ − 2 and ≤1), overweight (>1 and ≤2), and obesity (>2) (27). Because shorter completion times indicate better performance for the 50-m dash and endurance running, these two components were reverse-coded when calculating the PFI. The overall PFI was calculated as: $\begin{array}{l} \mathrm{PFI}=Z_{\left(\mathrm{FVC}\right)}+Z_{\left(\text{StandingLongJump}\right)}+Z_{\left(\mathrm{Sit}-\text{and}-\text{Reach}\right)}\\ +Z_{\left(\text{BodyMuscularStrength}\right)}+Z_{\left(\text{Handgrip}\right)}-Z_{\left(50-\text{mDash}\right)}\\ -Z_{\left(\text{EnduranceRunning}\right)} \end{array}$ The PFI represents a relative composite measure of overall physical fitness standardized against the 2005 sex- and age-specific reference distribution, with higher PFI values indicating better overall physical fitness.

### 2.3 Statistics

Continuous variables are presented as mean ± standard deviation (SD), and categorical variables are presented as frequencies and percentages. Univariate analysis of variance (ANOVA) and chi-square tests were conducted to compare physical fitness and nutritional status across survey years, supplemented by trend analyses. Fractional polynomial regression was employed to evaluate the nonlinear association between BMI Z-score and PFI. PFI was treated as the dependent variable and BMI Z-score was treated as the independent variable. Fractional polynomial models were fitted using linear regression, and the optimal transformation of BMI Z-score was selected from 44 candidate models based on model fit criteria. Analyses were conducted separately for each survey year (2005, 2010, 2014, and 2019). No additional covariates were included in these models. The corresponding model parameters are presented in the Supplementary Table S4. Additionally, generalized additive models (GAMs) were used to assess the associations between PFI and adverse nutritional status (thinness, overweight, and obesity), with results expressed as odds ratios (ORs). Thinness, overweight, and obesity were modeled separately as binary outcomes, with PFI as the main predictor. GAMs were fitted using the mgcv package in R with a binomial distribution and logit link function. Penalized regression splines were used to model nonlinear relationships between PFI and nutritional-status outcomes, and year-specific smooth functions were applied to evaluate temporal patterns. Age and sex were included as covariates in the overall analyses, whereas sex was excluded from sex-stratified analyses. Odds ratios were obtained by exponentiating the predicted log-odds from the GAM models, with the corresponding reference value specified for interpretation. All statistical tests were two-sided, and *p <* 0.05 was considered statistically significant. Statistical analyses and graphical visualization were performed using StataMP 17, Origin 2024 (OriginLab Corporation), and R software. The CNSSCH adopted a stratified random cluster sampling design according to the official implementation procedures issued by the Ministry of Education of the People’s Republic of China. However, sampling weights, cluster identifiers (e.g., school or class identifiers), and stratum identifiers were unavailable in the dataset provided to our research team. Therefore, survey-specific procedures accounting for the complex sampling design were not applied in the present analyses.

## 3 Results

Across the four surveys conducted in 2005, 2010, 2014, and 2019, a total of 9,237 Tibetan children and adolescents were included in the final analysis. The proportions of boys and girls remained generally comparable across survey years. From 2005 to 2019, height and weight showed continuous increases, whereas BMI decreased slightly in 2010 and subsequently increased in 2014 and 2019. The prevalence of thinness increased from 2005 to 2010 and declined thereafter in 2014 and 2019. The proportion of normal-weight participants decreased in 2010 and gradually returned to a level close to that observed in 2005. In contrast, the prevalence of overweight increased across the four survey years, whereas the prevalence of obesity increased until 2014 and slightly declined in 2019. Compared with the 2005 survey, the mean PFI increased by 4.91 standardized units in 2019 (*Z* = 1644.89, *P_trend_* < 0.001) (Table 1). In analyses stratified by nutritional status, standardized height and weight scores increased across all nutritional-status groups. BMI Z-scores remained relatively stable among the thinness group but increased overall among the normal-weight, overweight, and obesity groups (Supplementary Table S5).

**Table 1:** Nutritional status and physical fitness of Tibetan children and adolescents aged 7–18 years, 2005–2019.

Variable	2005	2010	2014	2019	*F/χ^2^*	*P*	*P_trend_*
Sample size	2,275	2,544	2,297	2,121	–	–	–
Sex							
Boys	1,127 (49.5%)	1,253 (49.3%)	1,134 (49.4%)	1,044 (49.2%)	–	–	–
Girls	1,148 (50.5%)	1,291 (50.7%)	1,163 (50.6%)	1,077 (50.8%)	–	–	–
Age groups (years)							
7–9	582 (25.6%)	653 (25.7%)	581 (25.3%)	417 (19.7%)	–	–	–
10–12	582 (25.6%)	661 (26%)	567 (24.7%)	572 (27%)	–	–	–
13–15	546 (24.0%)	643 (25.3%)	579 (25.2%)	544 (25.6%)	–	–	–
16–18	565 (24.8%)	587 (23.1%)	570 (24.8%)	588 (27.7%)	–	–	–
Height (cm)	144.95 ± 16.53	147.17 ± 16.05	148.86 ± 15.88	150.52 ± 15.0	70.62	<0.001	<0.001
Weight (kg)	38.51 ± 12.49	39.07 ± 12.84	40.85 ± 12.79	43.09 ± 12.1	75.43	<0.001	<0.001
BMI (kg/m^2^)	17.77 ± 2.67	17.48 ± 2.83	17.91 ± 2.83	18.57 ± 2.68	66.15	<0.001	<0.001
Nutritional status							
Normal weight	1884 (82.8%)	1901 (74.7%)	1804 (78.5%)	1752 (82.6%)	228.00	<0.001	<0.001^*^
Thinness	227 (10.0%)	425 (16.7%)	255 (11.1%)	101 (4.8%)			
Overweight	125 (5.5%)	165 (6.5%)	176 (7.7%)	213 (10.0%)			
Obese	39 (1.7%)	53 (2.1%)	62 (2.7%)	55 (2.6%)			
Physical fitness status							
PFI	Ref	0.42 ± 4.75	2.22 ± 4.74	4.91 ± 4.79	632.54	<0.001	<0.001
Forced vital capacity (mL)	1447.06 ± 601.37	1504.61 ± 678.96	1864.29 ± 878.05	2269.32 ± 779.56	699.06	<0.001	<0.001
Standing long jump (cm)	158.20 ± 39.63	155.7 ± 35.57	159.58 ± 36.71	160.13 ± 37.61	7.12	<0.001	0.022
Sit-and-reach (cm)	7.57 ± 5.86	7.59 ± 5.96	6.42 ± 5.95	8.90 ± 6.09	62.58	<0.001	<0.001
50 m dash (s)	9.83 ± 1.65	9.85 ± 1.46	9.70 ± 1.42	9.45 ± 1.49	36.10	<0.001	<0.001
Handgrip	21.16 ± 10.90	21.06 ± 10.32	21.90 ± 10.30	25.31 ± 11.42	75.59	<0.001	<0.001
Body muscle strength							
Oblique body pull-ups (boys aged 7–12 years)	46.86 ± 14.02	22.48 ± 9.56	31.13 ± 16.04	33.06 ± 18.24	643.02	<0.001	<0.001
Pull-ups (boys aged 13–18 years)	5.58 ± 3.98	5.15 ± 4.23	7.07 ± 5.31	10.63 ± 7.73	86.12	<0.001	<0.001
Sit-ups (girls aged 7–18 years)	14.65 ± 8.57	25.70 ± 10.86	25.17 ± 10.27	25.02 ± 11.77	580.22	<0.001	<0.001
Endurance running (s)							
8 × 50 m shuttle run (boys aged 7–12 years)	130.48 ± 20.32	128.95 ± 13.76	127.87 ± 14.72	131.45 ± 16.60	11.75	<0.001	<0.001^**^
1,000 m run (boys aged 13–18 years)	285.58 ± 37.66	290.67 ± 38.56	275.74 ± 36.06	267.16 ± 34.56	51.36	<0.001	<0.001^**^
800 m run (girls aged 13–18 years)	273.01 ± 31.15	289.08 ± 36.73	278.00 ± 32.45	263.84 ± 28.37	63.28	<0.001	<0.001^**^

* Indicates chi-square trend test, ** indicates quadratic trend test.

From 2005 to 2019, FVC increased continuously, whereas standing long jump, sit-and-reach, 50-m dash, and endurance running showed fluctuations across survey years without a consistent increasing or decreasing pattern (Figure 2; Supplementary Table S6). The normal-weight group generally exhibited the highest PFI across all survey years. The PFI values of the thinness, overweight, and obesity groups reached their highest levels in 2019. For individual fitness components, the thinness group generally showed lower FVC, sit-and-reach, and handgrip strength values. Compared with the normal-weight group, the overweight and obesity groups had higher FVC and handgrip strength values but lower standing long jump performance and longer completion times for the 50-m dash and endurance running.

**Figure 2:**
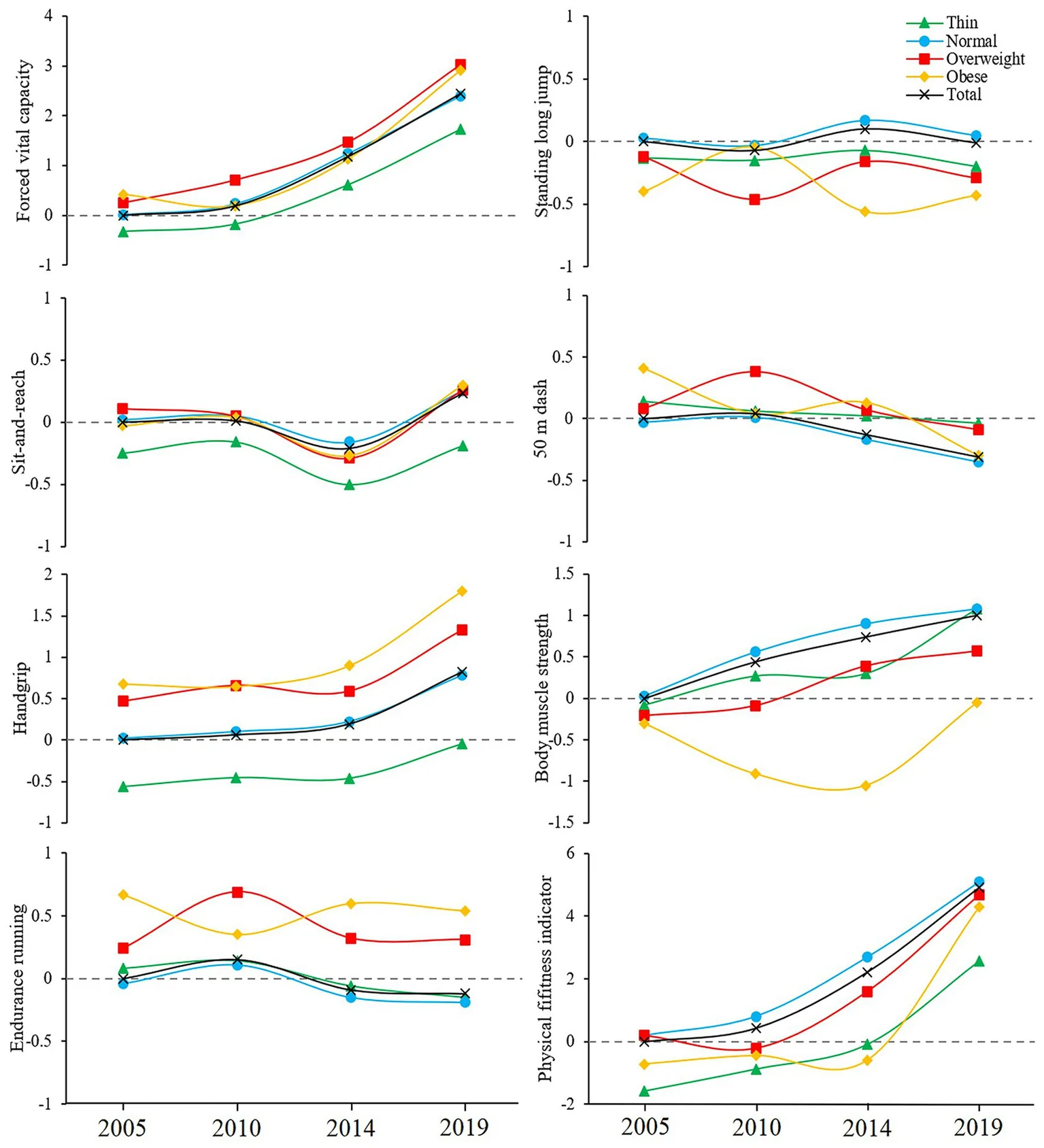
Trends in physical fitness across nutritional status groups, 2005–2019.

In sex-stratified analyses, PFI among boys decreased in 2010 and subsequently increased in 2014 and 2019, whereas PFI among girls increased consistently across survey years. Among boys, the largest increase in PFI from 2005 to 2019 was observed in the normal-weight group, increasing from 0.27 to 4.31 (*Z* = 500.32, *P_trend_* < 0.001). Among girls, the largest increase was observed in the obesity group, increasing from −0.15 to 6.71 (*Z* = 30.49, *P_trend_* < 0.001) (Supplementary Table S7; Supplementary Figure S1). Across age groups, PFI values were higher in 2019 than in 2005, although the trajectories differed among age groups (Supplementary Figure S2). FVC showed greater increases in the 10–12-year and 13–15-year groups, whereas muscular strength showed greater increases among girls and in the 13–15-year and 16–18-year groups (Supplementary Figures S3, S4).

Fractional polynomial regression analyses showed an inverted U-shaped association between BMI Z-score and PFI in each survey year. As BMI Z-score increased from lower levels, PFI gradually increased and reached its highest value within the intermediate BMI Z-score range, followed by a decline at higher BMI Z-score levels. PFI was lower among children and adolescents with BMI Z-scores at both the lower and higher ends of the distribution compared with those in the intermediate range (Figure 3). The fractional polynomial regression models showed statistically significant associations in all four survey years (all *p* < 0.001; Supplementary Table S4). The supplementary analyses stratified by survey year showed the same pattern (Supplementary Figure S5). Except for the 50-m dash and endurance running, most individual fitness components also showed an inverted U-shaped relationship with BMI Z-score (Supplementary Figure S6).

**Figure 3:**
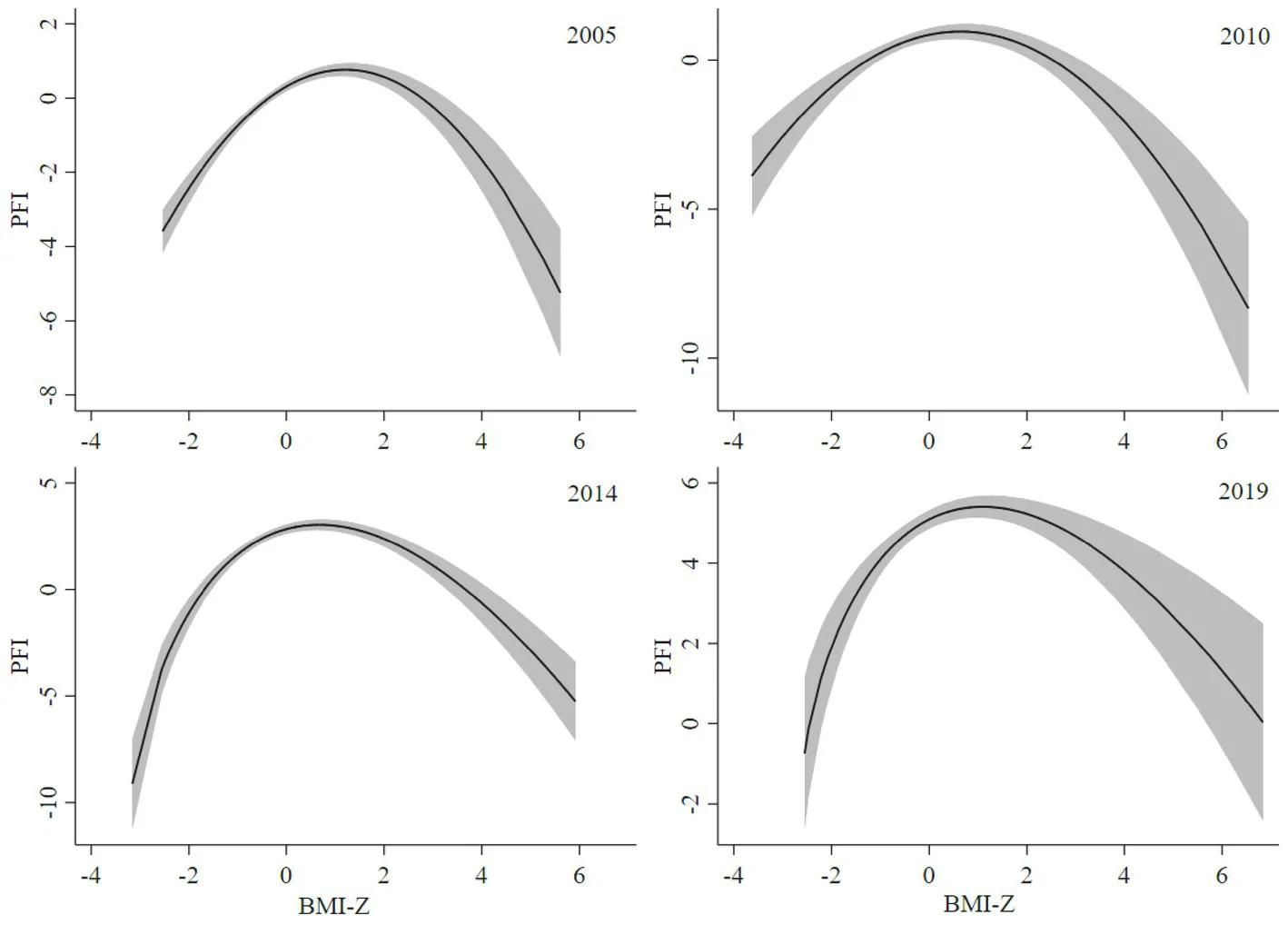
Association between PFI and BMI Z-score, 2005–2019. The shaded areas represent the 95% confidence intervals of the fitted curves.

Analysis of the associations between PFI and malnutrition categories, expressed as prevalence and *ORs* (Figure 4), showed that children and adolescents with better physical fitness (PFI > 0) generally had lower prevalence and lower odds of thinness, overweight, and obesity. In contrast, among those with poorer physical fitness (PFI < 0), the prevalence and odds of thinness increased markedly as PFI decreased, whereas those of overweight and obesity increased more gradually. By 2019, children and adolescents with poorer physical fitness exhibited higher odds of thinness than of overweight and obesity. Using PFI = −5 and PFI = 5 as representative examples, at PFI = −5, the estimated *ORs* for thinness were approximately 1.53 (*95% CI*: 1.37–1.68), 1.78 (1.60–1.95), 1.88 (1.68–2.09), and 1.84 (1.66–2.03) in 2005, 2010, 2014, and 2019, respectively, whereas the corresponding *ORs* for overweight and obesity were 1.10 (1.00–1.20), 1.32 (1.15–1.49), 1.75 (1.51–2.00), and 1.16 (1.03–1.29). At PFI = 5, the estimated *ORs* for thinness declined to approximately 0.43 (0.32–0.55), 0.16 (0.05–0.28), 0.25 (0.08–0.41), and 0.32 (0.14–0.50), respectively, while the corresponding *ORs* for overweight and obesity were 0.90 (0.80–1.00), 0.62 (0.49–0.74), 0.49 (0.31–0.67), and 0.78 (0.66–0.91).

**Figure 4:**
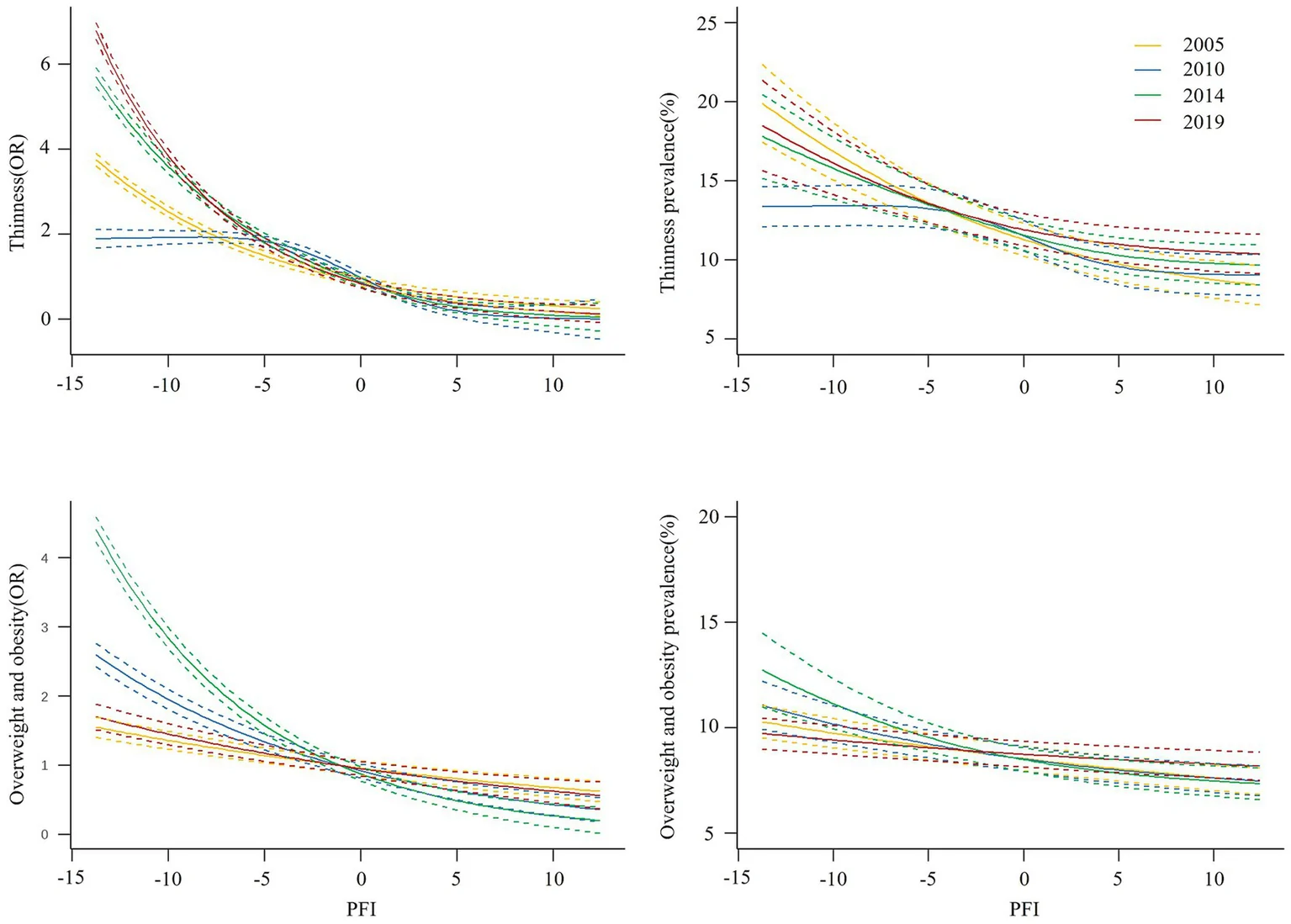
Prevalence of malnutrition categories and odds ratios (*ORs*) in relation to PFI. Dashed lines represent the 95% confidence intervals of the estimated odds ratios and prevalence curves.

## 4 Discussion

This study analyzed data from 9,237 Tibetan children and adolescents aged 7–18 years across four survey waves between 2005 and 2019 to examine long-term trends in physical fitness and explore the association between physical fitness and nutritional status. The findings showed an overall increase in physical fitness over time. Meanwhile, the prevalence of thinness increased initially and subsequently declined, whereas the prevalence of overweight/obesity increased over the study period. An inverted U-shaped relationship was observed between PFI and BMI Z-score, indicating that children and adolescents with thinness or overweight/obesity had lower physical fitness than those with normal weight. In addition, lower PFI values were associated with higher odds of both thinness and overweight/obesity, with the increase in the odds of thinness being more pronounced.

Physical fitness showed a significant improvement among Tibetan children and adolescents during the study period. The present study demonstrated that the PFI increased continuously from 2005 to 2019, indicating an overall enhancement in physical fitness over the study period. This finding is consistent with a previous CNSSCH-based study that investigated geographical variations in physical fitness among Chinese children and adolescents from 2005 to 2014. That study reported an overall improvement in PFI and showed that Tibet was among the regions with the largest reductions in the proportion of students with low PFI (28). However, previous studies covering longer historical periods have reported different trends. Fühner et al. (11), based on studies conducted between 1972 and 2015, reported a global decline in physical fitness among children and adolescents, particularly in cardiorespiratory endurance from the mid-1980s to the early 2010s. Similarly, Dong et al. (29) found that PFI among Chinese children and adolescents increased before 1995 but declined thereafter through 2014, while a comparable downward trend after the mid-1990s was also observed among children and adolescents in Xinjiang (30). These differences may be related to variations in study periods, geographical regions, and population characteristics. The improvement observed among Tibetan children and adolescents from 2005 to 2019 occurred during a period of rapid socioeconomic development and increasing attention to youth physical health in Tibet. Previous studies have reported relatively high levels of physical activity among Tibetan youth (19), and culturally embedded activities such as Guozhuang and Tibetan dance may provide additional opportunities for physical activity participation (31, 32). In addition, improvements in school-based physical education programs and health promotion policies may have contributed to the observed increase in physical fitness (33–35). Overall, the improvement in physical fitness observed in this population may reflect the combined influence of multiple social, environmental, and behavioral changes during this period. However, the specific contributions of these factors could not be determined because related information was not collected in the present study.

The nutritional status of Tibetan children and adolescents also underwent substantial changes during the study period. In the present study, the prevalence of thinness decreased overall from 10.0% in 2005 to 4.8% in 2019, whereas the prevalence of overweight and obesity increased from 5.5 to 10.0% and from 1.7 to 2.6%, respectively. These findings are broadly consistent with previous evidence from Tibetan populations. Using data from the CNSSCH in 1985, 1995, 2000, and 2005, Ma et al. (22) reported that the nutritional status of Tibetan students improved over time, characterized by a decline in undernutrition but a continuous increase in overweight and obesity. The present findings suggest that the nutritional transition observed among Tibetan students before 2005 continued during the subsequent period. Similar transitions have also been documented among Chinese children and adolescents at the national level, with a gradual decrease in undernutrition accompanied by an increasing prevalence of overweight and obesity (29). However, the nutritional transition observed in the present study was not entirely linear, as thinness temporarily increased in 2010 before declining markedly thereafter, while obesity showed relatively little change between 2014 and 2019. These fluctuations may reflect the complex interaction between socioeconomic development, nutritional interventions, and lifestyle changes during different stages of nutritional transition. Improvements in living conditions and nutrition-related interventions may have contributed to the reduction in undernutrition among Tibetan children and adolescents (36, 37), whereas dietary changes and lifestyle transitions associated with modernization may have contributed to the increasing prevalence of overweight (38, 39). However, these potential explanations should be interpreted cautiously because dietary intake, physical activity, and other lifestyle-related factors were not directly assessed in the present study.

The present study further demonstrated a consistent inverted U-shaped association between BMI Z-score and PFI across all four survey waves, with normal-weight children and adolescents exhibiting the highest overall physical fitness, whereas both thinness and overweight/obesity were associated with lower PFI. This finding is consistent with previous studies showing that physical fitness is generally optimized among children and adolescents with normal weight status. Zhang et al. (40) reported an inverted U-shaped relationship between BMI and PFI among Tibetan children and adolescents living at different altitudes, while similar nonlinear associations have also been observed among Chinese (41), Xinjiang (30), Chinese and Japanese (17), and Taiwanese (13) youth. Dong et al. (29) further demonstrated that both underweight and overweight/obese Chinese children and adolescents had poorer physical fitness compared with their normal-weight peers. However, previous studies have suggested that the strength and pattern of the association between BMI and physical fitness may vary according to sex, age, and geographical background. For example, Zhang et al. (40) reported differences in the BMI–PFI relationship across altitude regions, while Huang (13) and Kwiecinski et al. (42) identified age- and sex-related variations in the relationship between BMI and physical fitness. Interestingly, although overweight and obese children and adolescents generally showed lower PFI values than their normal-weight peers, they also experienced substantial improvements in physical fitness during the study period. According to the supplementary analysis, PFI increased by 4.49 units in the overweight group and 5.02 units in the obese group from 2005 to 2019, indicating that improvements in physical fitness occurred across all nutritional categories. The relatively larger changes observed among overweight and obese groups may partly reflect their lower baseline PFI values in 2005, leaving greater potential for improvement over time. In addition, the observed improvements across multiple physical fitness components included in the composite PFI may have collectively contributed to the overall increase in PFI. Nevertheless, despite these improvements, overweight and obese children and adolescents continued to exhibit lower overall physical fitness than normal-weight peers in 2019. Previous studies have suggested that excess body weight may increase mechanical burden during weight-bearing activities and negatively affect exercise performance, particularly endurance-related and mobility-related fitness components (43). In contrast, thin children and adolescents may experience limitations associated with insufficient nutritional status, which may negatively influence physical fitness (44). However, these potential mechanisms should be interpreted cautiously because body composition, dietary intake, and physical activity were not directly assessed in the present study.

This study used a large sample of Tibetan children and adolescents to assess long-term trends in physical fitness and to examine its relationship with nutritional status, providing valuable evidence for understanding physical fitness development in this population. Nevertheless, several limitations should be acknowledged. First, the four CNSSCH survey waves did not follow the same individuals over time. Therefore, the observed temporal changes in physical fitness reflect population-level trends across survey years rather than within-person longitudinal changes, and the observed associations between physical fitness and nutritional status cannot be interpreted as causal relationships. Second, the surveys did not collect information on several potentially important factors, including dietary intake, physical activity, socioeconomic conditions, and the home environment. Because these factors could not be accounted for in the analyses, residual confounding cannot be excluded. Third, substantial socioeconomic and environmental changes occurred in Tibet during the study period. Because these factors were not measured and could not be accounted for in the analyses, residual confounding cannot be excluded. Fourth, although the CNSSCH adopted a nationally standardized sampling framework, the Tibetan sample was drawn mainly from schools in and around Lhasa, and the distribution of urban and rural participants was not balanced across survey waves. Consequently, separate urban–rural trend analyses were not performed because the two subsamples were not sufficiently comparable across survey years. Furthermore, because sampling weights and sampling design variables (including cluster and stratum identifiers) were unavailable in the dataset provided to our research team, the complex sampling structure of the CNSSCH could not be fully incorporated into the analyses. This limitation may have affected the estimation of standard errors and confidence intervals and should be considered when interpreting the statistical uncertainty of the findings. Finally, although anthropometric and physical fitness measurements were conducted according to standardized national procedures, with qualified examiners and equipment checking and calibration, some between-wave variation related to examiners, equipment, survey timing, or implementation conditions over the study period cannot be completely ruled out.

## 5 Conclusion

Although physical fitness improved among Tibetan children and adolescents over the study period, both thinness and overweight/obesity were associated with less favorable physical fitness. These findings suggest that maintaining an appropriate weight status may be important for promoting favorable physical fitness development among children and adolescents.

## Data Availability

The participant-level Tibetan CNSSCH data used in this study are not publicly available. The data used in the present study are under the custodianship of Tibet University for Nationalities and were managed by co-author ML, who was responsible for the preparation of physical fitness and health reports for the Education Department of the Tibet Autonomous Region. The use of these data for the present study was authorized by the Education Department of the Tibet Autonomous Region. The authors are not authorized to redistribute the participant-level data to third parties. Researchers interested in accessing the data should submit a request to the Education Department of the Tibet Autonomous Region, and access is subject to authorization by the relevant authority. Requests to access these datasets should be directed to YS, sunyi0084@163.com.
