# Supplementary material for: Secular trends in physical fitness and nutritional status among Tibetan children and adolescents from 2005 to 2019: a secondary analysis of four CNSSCH surveys
Source: Front Nutr. 2026 Sep 30;13:1891556. doi: 10.3389/fnut.2026.1891556 (PMC13661342; doi:10.3389/fnut.2026.1891556)
Supplement: Supplementary file 1 [file Supplementary_file_1.docx]

**Supplementary**

**Calculation of the Physical Fitness Index (PFI)**

The Physical Fitness Index (PFI) was constructed as a composite measure of overall physical fitness based on the approach described by Huang and Malina^[1]^. The PFI incorporated seven physical fitness components: forced vital capacity (FVC), standing long jump, sit-and-reach, handgrip strength, body muscular strength, 50-m dash, and endurance running. FVC, standing long jump, sit-and-reach, handgrip strength, and the 50-m dash were assessed in both boys and girls aged 7–18 years. The specific tests used to assess body muscular strength and endurance performance varied according to age and, where applicable, sex, in accordance with the standardized CNSSCH testing procedures. Body muscular strength was assessed by oblique body pull-ups in boys aged 7–12 years, standard pull-ups in boys aged 13–18 years, and 1-min sit-ups in girls aged 7–18 years. Endurance performance was assessed using the 8 × 50-m shuttle run in children aged 7–12 years and distance endurance running (1000 m for boys and 800 m for girls) in adolescents aged 13–18 years. The physical fitness components and their units of measurement were as follows: FVC (mL), standing long jump (cm), sit-and-reach (cm), handgrip strength (kg), body muscular strength (number of repetitions), 50-m dash (s), and endurance running (s).

To account for differences in the scales and units of the individual physical fitness measures, each component was standardized separately by sex and single-year age using the corresponding reference distribution from the 2005 CNSSCH survey. The 2005 survey was selected as the fixed reference because it was the earliest survey wave included in the present study and provided a common baseline for evaluating changes in physical fitness across subsequent survey years. The same 2005 reference values were therefore applied to participants in the 2005, 2010, 2014, and 2019 surveys.

For each physical fitness component, the standardized score was calculated as:

$$Z=\frac{X-M_{2005}}{SD_{2005}}$$

where$X$ represents the individual measurement, $M_{2005}$represents the sex- and age-specific median of the corresponding indicator in the 2005 survey, and $SD_{2005}$represents the corresponding sex- and age-specific standard deviation. The sex- and age-specific 2005 reference values used in the calculation are provided in Supplementary Table S3.

For FVC, standing long jump, sit-and-reach, handgrip strength, and body muscular strength, higher values indicate better physical fitness; therefore, the standardized scores were retained in their original direction. In contrast, the 50-m dash and endurance-running tests were measured as completion time, for which lower values indicate better performance. The standardized scores for these two components were therefore multiplied by −1 so that higher standardized scores consistently represented better performance across all PFI components.

The PFI was then calculated by combining the standardized scores of the seven physical fitness components:

$$PFI=Z_{FVC}+Z_{standing\text{ }long\text{ }jump}+Z_{sit-and-reach}+Z_{handgrip\text{ }strength}+Z_{body\text{ }muscular\text{ }strength}-Z_{50-m\text{ }dash}-Z_{endurance\text{ }running}$$

Accordingly, a higher PFI indicates better overall physical fitness, whereas a lower PFI indicates poorer overall physical fitness. Because all seven component measures were required for calculation of the PFI, participants with a missing value for any required component were excluded from the PFI analysis; no missing component values were imputed.

**Worked example of PFI calculation**

As an illustrative example, a 16-year-old boy from the 2019 survey had an FVC of 3973 mL, standing long jump of 245 cm, sit-and-reach of 14.8 cm, handgrip strength of 45.9 kg, body muscular strength of 10 repetitions, 50-m dash time of 7.3 s, and endurance-running time of 278.22 s.

Using the sex- and age-specific median and standard deviation from the 2005 reference population in Table S3, each physical fitness component was standardized according to the formula presented above.

For FVC:

$$Z_{FVC}=\frac{3973-2279.0}{580.7}=2.917$$

For standing long jump:

$$Z_{standing\text{ }long\text{ }jump}=\frac{245-222.0}{17.2}=1.337$$

For sit-and-reach:

$$Z_{sit-and-reach}=\frac{14.8-11.4}{5.9}=0.576$$

For handgrip strength:

$$Z_{handgrip}=\frac{45.9-37.4}{6.8}=1.250$$

For body muscular strength:

$$Z_{body\text{ }muscular\text{ }strength}=\frac{10-5.0}{3.0}=1.667$$

For the 50-m dash:

$$Z_{50-m\text{ }dash}=\frac{7.3-7.8}{0.6}=-0.833$$

For endurance running:

$$Z_{endurance\text{ }running}=\frac{278.22-261.8}{29.0}=0.566$$

Because shorter completion times indicate better performance for the 50-m dash and endurance running, these two standardized scores were reverse-coded when calculating the PFI. Therefore:

$$PFI=2.917+1.337+0.576+1.250+1.667-\left( -0.833 \right)-0.566$$

$$PFI=8.014$$

Thus, the participant had a PFI of 8.014. A higher PFI represents better overall physical fitness relative to the sex- and age-specific 2005 reference distribution.

Table S1 Sample Size and Percentage of Anthropometric and Physical Fitness Measures, 2005-2019

| **Survey year** | **2005** | **2010** | **2014** | **2019** |
| --- | --- | --- | --- | --- |
| Total sample size | 2275 | 2544 | 2297 | 2121 |
| **Anthropometric measurement, n (%)** |  |  |  |  |
| Height | 2275（100%） | 2544（100%） | 2297（100%） | 2121（100%） |
| Weight | 2275（100%） | 2544（100%） | 2297（100%） | 2121（100%） |
| **Physical fitness measurement, n (%)** |  |  |  |  |
| Forced Vital Capacity | 2275（100%） | 2544（100%） | 2297（100%） | 2121（100%） |
| Standing Long Jump | 2275（100%） | 2544（100%） | 2297（100%） | 2121（100%） |
| Sit and Reach | 2275（100%） | 2544（100%） | 2297（100%） | 2121（100%） |
| 50 Meter Dash | 2275（100%） | 2544（100%） | 2297（100%） | 2121（100%） |
| Body Muscle Strength | 2275（100%） | 2544（100%） | 2297（100%） | 2121（100%） |
| Handgrip | 2275（100%） | 2544（100%） | 2297（100%） | 2121（100%） |
| Endurance running | 2275（100%） | 2544（100%） | 2297（100%） | 2121（100%） |

Note: n (%), the sample size and percentage completing each physical fitness measurement.

Table S2 Parameters for the median values and standard deviations of anthropometric indicators in the 2005 reference population

| **Age/Year** | **Sample size** | **Height (cm)** | **Weight (kg)** | **BMI (kg/m^2^)** |
| --- | --- | --- | --- | --- |
| **Boys** |  |  |  |  |
| 7 | 97 | 117.7（5.5） | 21.2（3.0） | 15.4（1.2） |
| 8 | 95 | 124.9（6.0） | 24.6（3.8） | 15.8（1.9） |
| 9 | 97 | 132.1（6.4） | 28.2（4.2） | 16.0（1.5） |
| 10 | 97 | 132.1（4.9） | 28.5（4.6） | 16.1（1.9） |
| 11 | 96 | 140.3（6.5） | 32.0（5.0） | 16.3（1.7） |
| 12 | 96 | 142.5（7.9） | 33.9（5.7） | 16.7（1.6） |
| 13 | 83 | 145.6（8.3） | 36.8（7.7） | 17.0（2.9） |
| 14 | 87 | 157.8（7.9） | 45.2（6.6） | 17.6（1.6） |
| 15 | 96 | 162.2（7.1） | 48.3（7.0） | 18.2（1.9） |
| 16 | 93 | 167.0（5.3） | 52.8（5.8） | 19.0（1.8） |
| 17 | 95 | 168.9（5.1） | 55.3（5.0） | 19.3（1.7） |
| 18 | 95 | 168.1（5.0） | 55.7（6.0） | 19.5（1.8） |
| **Girls** |  |  |  |  |
| 7 | 94 | 117.0（6.7） | 20.6（3.6） | 15.1（1.3） |
| 8 | 100 | 124.6（6.6） | 24.0（4.3） | 15.1（1.8） |
| 9 | 99 | 130.5（7.3） | 26.8（5.8） | 15.5（2.2） |
| 10 | 100 | 134.5（5.6） | 28.5（4.8） | 15.8（2.0） |
| 11 | 96 | 141.5（6.0） | 33.1（4.8） | 16.5（1.8） |
| 12 | 97 | 145.0（6.3） | 33.8（6.7） | 16.5（2.2） |
| 13 | 93 | 147.0（7.0） | 40.7（7.2） | 18.6（3.1） |
| 14 | 96 | 153.2（7.3） | 47.0（8.4） | 19.6（3.0） |
| 15 | 91 | 155.7（5.0） | 47.1（6.1） | 19.7（2.2） |
| 16 | 91 | 156.9（5.6） | 50.9（5.4） | 20.4（2.4） |
| 17 | 96 | 157.6（4.7） | 51.1（5.8） | 20.4（2.1） |
| 18 | 95 | 156.6（4.8） | 50.4（5.0） | 20.1（2.0） |

Note: Values are presented as median (standard deviation). Sex- and age-specific values from the 2005 survey were used as the reference parameters for standardization of height, weight, and BMI in subsequent survey years.

Table S3 Parameters for the median values and standard deviations of each physical fitness item in the 2005 reference population

| **Age/Year** | **Sample**  **size** | **Forced Vital**  **Capacity(ml)** | **Standing Long**  **Jump (cm)** | **Sit and**  **Reach (cm)** | **Handgrip** | **50 Meters Dash (s)** | **Body Muscle Strength*** | **Endurance**  **Running (s)*** |
| --- | --- | --- | --- | --- | --- | --- | --- | --- |
| **Boys** |  |  |  |  |  |  |  |  |
| 7 | 97 | 942.0(195.9) | 112.0(15.9) | 5.4(4.5) | 8.9(2.1) | 12.3(1.0) | 31.0(15.4) | 139.8(21.4) |
| 8 | 95 | 996.0(268.7) | 130.0(20.3) | 6.0(4.4) | 11.2(3.0) | 10.8(1.0) | 44.0(12.05) | 136.0(21.8) |
| 9 | 97 | 1045.0(262.9) | 144.0(21.9) | 7.3(4.7) | 13.6(3.3) | 10.4(1.1) | 47.0(9.73) | 131.6(19.8) |
| 10 | 97 | 1113.0(213.6) | 142.0(13.2) | 4.2(4.8) | 14.3(2.5) | 9.9(1.0) | 50.0(12.88) | 117.4(11.1) |
| 11 | 96 | 1229.0(306.4) | 159.0(16.5) | 4.3(4.7) | 16.5(3.9) | 9.7(0.8) | 52.5(11.83) | 116.6(10.4) |
| 12 | 96 | 1384.5(306.2) | 168.0(18.7) | 6.3(5.0) | 18.5(4.9) | 9.2(1.0) | 54.0(9.31) | 111.5(16.7) |
| 13 | 83 | 1427.0(333.9) | 175.0(15.1) | 6.7(6.2) | 21.6(5.6) | 9.1(0.8) | 2.0(3.23) | 313.0(31.2) |
| 14 | 87 | 1650.0(404.4) | 187.0(16.7) | 4.6(5.1) | 27.1(6.9) | 8.5(1.0) | 4.0(3.5) | 309.9(36.9) |
| 15 | 96 | 1890.0(440.5) | 197.0(18.8) | 6.0(5.7) | 31.4(7.0) | 8.1(0.8) | 4.0(2.6) | 272.2(30.7) |
| 16 | 93 | 2279.0(580.7) | 222.0(17.2) | 11.4(5.9) | 37.4(6.8) | 7.8(0.6) | 5.0(3.0) | 261.8(29.0) |
| 17 | 95 | 2579.0(464.3) | 232.0(16.3) | 11.0(6.7) | 42.7(5.1) | 7.5(0.4) | 8.0(4.2) | 296.6(42.5) |
| 18 | 95 | 2547.0(527.9) | 235.0(17.8) | 12.8(5.7) | 43.2(5.3) | 7.5(0.6) | 8.0(4.3) | 263.7(26.1) |
| **Girls** |  |  |  |  |  |  |  |  |
| 7 | 94 | 856.5(202.3) | 96.5(13.5) | 8.5(4.3) | 6.9(2.4) | 12.9(1.3) | 5.0(7.3) | 147.6(19.3) |
| 8 | 100 | 911.0(241.5) | 110.0(15.5) | 7.6(4.4) | 9.6(2.7) | 11.5(1.0) | 6.0(4.8) | 146.2(20.5) |
| 9 | 99 | 887.0(226.1) | 125.0(18.3) | 6.8(4.6) | 12.1(3.1) | 11.0(1.1) | 10.0(5.9) | 141.4(16.2) |
| 10 | 100 | 1045.0(236.0) | 133.0(12.9) | 7.9(4.8) | 13.5(2.5) | 10.4(1.0) | 13.0(5.9) | 123.2(14.1) |
| 11 | 96 | 1123.0(250.7) | 145.0(14.3) | 6.7(5.9) | 15.6(3.3) | 10.1(0.8) | 14.0(5.2) | 120.6(14.4) |
| 12 | 97 | 1137.0(274.2) | 149.0(21.5) | 6.4(5.2) | 17.0(4.7) | 9.9(1.0) | 13.0(4.9) | 123.0(15.1) |
| 13 | 93 | 1466.0(298.3) | 151.0(18.4) | 8.4(5.0) | 20.7(4.2) | 9.8(1.1) | 14.0(7.4) | 276.6(31.2) |
| 14 | 96 | 1381.5(285.4) | 149.0(16.7) | 6.7(6.3) | 22.2(5.1) | 9.7(0.8) | 17.0(6.0) | 278.7(33.4) |
| 15 | 91 | 1545.0(365.7) | 154.0(18.1) | 8.9(6.7) | 24.7(3.7) | 9.4(0.8) | 16.0(4.7) | 257.1(29.8) |
| 16 | 91 | 1578.0(406.1) | 160.0(20.5) | 8.4(5.1) | 25.9(5.8) | 8.6(0.9) | 30.0(8.7) | 276.3(28.7) |
| 17 | 96 | 1723.5(333.8) | 170.0(20.5) | 10.6(7.0) | 28.2(4.7) | 9.6(0.8) | 19.0(4.7) | 269.3(31.8) |
| 18 | 95 | 1608.0(321.1) | 160.0(18.8) | 10.4(6.5) | 27.2(4.7) | 9.8(0.6) | 20.0(6.5) | 268.1(29.1) |

Note: Body Muscle Strength* included oblique body pull-ups (boys aged 7–12 y), pull-ups (boys aged 13–18 y), and one-minute sit-ups (girls aged 7–18 y), and was assessed by calculating repetitions. Endurance Running* included the 50 m × 8 shuttle run (students aged 7–12 y), 1000 m run (boys aged 13–18 y), and 800 m run (girls aged 13–18 y). Because shorter times indicate better performance in the 50-meter dash and endurance running (i.e., these are “lower-is-better” indicators), their Z-scores were multiplied by -1 to maintain consistency in the directionality of the scores.

Table S4 Fractional polynomial regression parameters for Physical Fitness Index (PFI) and Body Mass Index (BMI) Z-scores

| **Parameters** | **Number of**  **screening** | **Best Powers** | | **β1**  **(95% CI)** | **β2**  **(95% CI)** | **Deviance difference** | **F values** | **P** | **R^2^** |
| --- | --- | --- | --- | --- | --- | --- | --- | --- | --- |
|  | **models** | **p** | **q** |  |  |  |  |  |  |
| **PFI and**  **BMI Z-Score** |  |  |  |  |  |  |  |  |  |
| 2005 | 44 | 1 | 2 | 2.33  (1.93, 2.73) | -0.31  (-0.37, -0.25) | 57.26 | 79.29 | <0.001 | 0.0337 |
| 2010 | 44 | 1 | 2 | 22.78  (16.11, 29.45) | -26.638  (-34.47, -18.81) | 41.31 | 22.67 | <0.001 | 0.0175 |
| 2014 | 44 | 1 | 1 | 7.56  (6.15, 8.97) | -3.22  (-3.83, -2.61) | 88.70 | 55.66 | <0.001 | 0.0463 |
| 2019 | 44 | 1 | 1 | 3.93  (2.60, 5.27) | -1.71  (-2.32, -1.11) | 21.69 | 17.53 | <0.001 | 0.0163 |

Note: Best powers of BMI Z-Score among 44 screening models. β1 and β2 represent the coefficients of the corresponding transformed BMI Z-score terms, presented with 95% confidence intervals.

Table S5 Trends in standardized height, weight, and BMI across nutritional status groups, 2005 to 2019

| Anthropometric Indicator | 2005 | 2010 | 2014 | 2019 | change  (2005-2019) | Z values | *P _trend_** |
| --- | --- | --- | --- | --- | --- | --- | --- |
| **Height** | Ref | 0.38 | 0.59 | 0.64 | 0.64 | 756.04 | <0.001 |
| Thinness | -0.17 | -0.09 | 0.33 | 0.38 | 0.55 | 53.45 | <0.001 |
| Normal weight | 0.01 | 0.41 | 0.60 | 0.60 | 0.59 | 599.00 | <0.001 |
| Overweight | 0.06 | 0.73 | 0.77 | 0.89 | 0.83 | 76.93 | <0.001 |
| Obese | 0.57 | 0.71 | 1.05 | 1.37 | 0.80 | 17.87 | 0.004 |
| **Weight** | Ref | 0.14 | 0.41 | 0.64 | 0.64 | 566.22 | <0.001 |
| Thinness | -1.15 | -1.09 | -0.88 | -0.82 | 0.33 | 52.80 | <0.001 |
| Normal weight | -0.01 | 0.17 | 0.33 | 0.46 | 0.47 | 422.70 | <0.001 |
| Overweight | 1.47 | 2.14 | 2.12 | 2.15 | 0.68 | 94.49 | <0.001 |
| Obese | 2.66 | 2.82 | 3.23 | 3.47 | 0.81 | 15.00 | <0.001 |
| **BMI** | Ref | -0.11 | 0.08 | 0.35 | 0.35 | 266.83 | <0.001 |
| Thinness | -1.38 | -1.51 | -1.43 | -1.36 | 0.02 | 21.75 | 0.435 |
| Normal weight | -0.02 | -0.09 | -0.02 | 0.14 | 0.16 | 116.24 | <0.001 |
| Overweight | 1.85 | 2.16 | 2.10 | 2.02 | 0.17 | 27.02 | 0.005 |
| Obese | 3.10 | 3.40 | 3.52 | 3.52 | 0.42 | 6.98 | 0.012 |

*Test for trend with survey year from 2005 to 2019, based on the continuous variables of height, weight, and BMI.

Table S6 Trends in standardized values of each physical fitness component, 2005 to 2019

| **Physical Fitness Component** | 2005 | 2010 | 2014 | 2019 | Change (2005-2019) | Z values | *P_trend_** |
| --- | --- | --- | --- | --- | --- | --- | --- |
| **Forced Vital Capacity** | Ref | 0.19 | 1.18 | 2.44 | 2.44 | 3739.58 | <0.001 |
| Thinness | -0.33 | -0.18 | 0.61 | 1.73 | 2.06 | 250.49 | <0.001 |
| Normal | 0.01 | 0.23 | 1.23 | 2.40 | 2.39 | 3033.54 | <0.001 |
| Overweight | 0.25 | 0.71 | 1.47 | 3.03 | 2.78 | 317.11 | <0.001 |
| Obese | 0.42 | 0.19 | 1.13 | 2.91 | 2.49 | 72.66 | <0.001 |
| **Standing Long Jump** | Ref | -0.07 | 0.10 | -0.01 | -0.01 | 25.15 | 0.24 |
| Thinness | -0.13 | -0.14 | -0.07 | -0.20 | 0.07 | 1.12 | 0.992 |
| Normal | 0.03 | -0.03 | 0.17 | 0.05 | 0.02 | 26.78 | 0.032 |
| Overweight | -0.12 | -0.46 | -0.16 | -0.29 | 0.17 | 8.66 | 0.302 |
| Obese | -0.40 | -0.04 | -0.56 | -0.43 | 0.03 | 5.96 | 0.448 |
| **Body Muscle Strength** | Ref | 0.44 | 0.74 | 1.00 | 1.00 | 391.68 | <0.001 |
| Thinness | -0.08 | 0.27 | 0.30 | 1.08 | 1.16 | 16.39 | <0.001 |
| Normal | 0.03 | 0.56 | 0.90 | 1.08 | 1.05 | 427.23 | <0.001 |
| Overweight | -0.21 | -0.94 | 0.39 | 0.57 | 0.78 | 15.08 | <0.001 |
| Obese | -0.30 | -0.91 | -1.05 | -0.05 | 0.25 | 15.25 | 0.836 |
| **Sit and Reach** | Ref | 0.01 | -0.21 | 0.23 | 0.23 | 175.76 | <0.001 |
| Thinness | -0.25 | -0.16 | -0.50 | -0.19 | 0.06 | 22.22 | 0.147 |
| Normal | 0.02 | 0.05 | -0.16 | 0.25 | 0.23 | 122.38 | <0.001 |
| Overweight | 0.11 | 0.05 | -0.29 | 0.05 | 0.06 | 23.04 | 0.602 |
| Obese | -0.03 | 0.04 | -0.27 | 0.30 | 0.33 | 7.77 | 0.331 |
| **50 Meter Dash** | Ref | 0.04 | -0.13 | -0.31 | -0.31 | 166.68 | <0.001 |
| Thinness | 0.14 | 0.06 | 0.02 | -0.04 | 0.18 | 8.39 | 0.061 |
| Normal | -0.03 | 0.01 | -0.17 | -0.35 | 0.32 | 145.11 | <0.001 |
| Overweight | 0.08 | 0.38 | 0.07 | -0.09 | 0.17 | 10.86 | 0.053 |
| Obese | 0.41 | 0.04 | 0.13 | -0.30 | 0.71 | 10.35 | 0.004 |
| **Endurance Running** | Ref | 0.15 | -0.09 | -0.12 | -0.12 | 95.74 | <0.001 |
| Thinness | 0.08 | 0.14 | -0.06 | -0.15 | 0.23 | 12.34 | 0.016 |
| Normal | -0.04 | 0.11 | -0.15 | -0.19 | 0.15 | 90.92 | <0.001 |
| Overweight | 0.24 | 0.69 | 0.32 | 0.31 | 0.07 | 12.78 | 0.004 |
| Obese | 0.67 | 0.35 | 0.60 | 0.54 | 0.13 | 5.24 | 0.693 |
| **PFI** | Ref | 0.42 | 2.22 | 4.91 | 4.91 | 1644.89 | <0.001 |
| Thinness | -1.57 | -0.87 | -0.08 | 2.57 | 4.14 | 76.05 | <0.001 |
| Normal | 0.19 | 0.80 | 2.70 | 5.10 | 4.91 | 1410.05 | <0.001 |
| Overweight | 0.19 | -0.22 | 1.60 | 4.68 | 4.49 | 106.16 | <0.001 |
| Obese | -0.72 | -0.45 | -0.59 | 4.30 | 5.02 | 34.47 | <0.001 |
| **Handgrip** | Ref | 0.06 | 0.19 | 0.82 | 0.82 | 851.60 | <0.001 |
| Thinness | -0.56 | -0.45 | -0.46 | -0.04 | 0.52 | 35.87 | <0.001 |
| Normal | 0.02 | 0.10 | 0.22 | 0.78 | 0.76 | 633.62 | <0.001 |
| Overweight | 0.47 | 0.66 | 0.59 | 1.33 | 0.86 | 70.328 | <0.001 |
| Obese | 0.68 | 0.65 | 0.90 | 1.80 | 1.12 | 34.41 | <0.001 |

*Test for trend with survey year from 2005 to 2019, based on the continuous variables of standardized values of each physical fitness component.

Table S7 Trends in the PFI in boys and girls, 2005 to 2019

| PFI | 2005 | 2010 | 2014 | 2019 | change  (2005-2019) | Z values | *P _trend_** |
| --- | --- | --- | --- | --- | --- | --- | --- |
| **Boys** | Ref | -0.90 | 1.04 | 4.06 | 4.06 | 635.12 | <0.001 |
| Thinness | -1.49 | -2.31 | -0.97 | 2.32 | 3.81 | 59.07 | <0.001 |
| Normal weight | 0.27 | -0.37 | 1.72 | 4.31 | 4.04 | 500.32 | <0.001 |
| Overweight | -0.10 | -1.78 | -0.33 | 3.62 | 3.72 | 43.41 | <0.001 |
| Obese | -1.73 | -2.34 | -1.98 | 1.98 | 3.71 | 13.65 | 0.004 |
| **Girls** | Ref | 1.71 | 3.37 | 5.74 | 5.74 | 1239.93 | <0.001 |
| Thinness | -1.71 | 0.98 | 1.47 | 3.09 | 4.80 | 61.35 | <0.001 |
| Normal weight | 0.11 | 1.83 | 3.55 | 5.84 | 5.73 | 1055.11 | <0.001 |
| Overweight | 0.36 | 1.71 | 3.53 | 5.57 | 5.21 | 91.21 | <0.001 |
| Obese | -0.15 | 2.68 | 2.56 | 6.71 | 6.86 | 30.49 | <0.001 |
| **Total** | Ref | 0.42 | 2.22 | 4.91 | 4.91 | 1644.89 | <0.001 |
| Thinness | -1.57 | -0.87 | -0.08 | 2.57 | 4.14 | 76.05 | <0.001 |
| Normal weight | 0.19 | 0.80 | 2.70 | 5.10 | 4.91 | 1410.05 | <0.001 |
| Overweight | 0.19 | -0.22 | 1.60 | 4.68 | 4.49 | 106.16 | <0.001 |
| Obese | -0.72 | -0.45 | -0.59 | 4.30 | 5.02 | 34.47 | <0.001 |

*Test for trend with survey year from 2005 to 2019, based on the continuous variables of PFI.

Figure S1 Trends in the PFI in students of different sexes, 2005 to 2019

Figure S2 Trends in the PFI in students of different age groups, 2005 to 2019

Figure S3 Trends in the standardized values of the specific fitness indicators in students from different sexes, 2005-2019

Figure S4 Trends in the standardized values of the specific fitness indicators in students from different age groups, 2005-2019

Figure S5 Linear regression analysis of BMI Z-score and PFI across survey years, 2005–2019

Note: The shaded areas represent the 95% confidence intervals of the fitted curves.

Figure S6 Associations between standardized values and BMI Z-scores for various physical fitness indicators, 2005-2019

**References**

[1] Huang YC, Malina RM. BMI and health-related physical fitness in Taiwanese youth 9-18 years. Med Sci Sports Exerc. 2007;39(4):701-708. <https://doi.org/10.1249/mss.0b013e31802f0512>.
